# Bat Rabies in France: A 24-Year Retrospective Epidemiological Study

**DOI:** 10.1371/journal.pone.0098622

**Published:** 2014-06-03

**Authors:** Evelyne Picard-Meyer, Emmanuelle Robardet, Laurent Arthur, Gérald Larcher, Christine Harbusch, Alexandre Servat, Florence Cliquet

**Affiliations:** 1 French Agency for Food, Environmental and Occupational Health & Safety (ANSES), Nancy Laboratory for Rabies and Wildlife, OIE Reference Laboratory for Rabies, European Union Reference Laboratory for Rabies, European Union Reference Laboratory for Rabies Serology, Malzeville, France; 2 Museum d'Histoire Naturelle de Bourges, Bourges, France; 3 SFEPM Chiroptera Group, Museum d'Histoire Naturelle de Bourges, Bourges, France; 4 Nature and Biodiversity Conservation Union, Perl-Kesslingen, Germany; University of Texas Medical Branch, United States of America

## Abstract

Since bat rabies surveillance was first implemented in France in 1989, 48 autochthonous rabies cases without human contamination have been reported using routine diagnosis methods. In this retrospective study, data on bats submitted for rabies testing were analysed in order to better understand the epidemiology of EBLV-1 in bats in France and to investigate some epidemiological trends. Of the 3176 bats submitted for rabies diagnosis from 1989 to 2013, 1.96% (48/2447 analysed) were diagnosed positive. Among the twelve recognised virus species within the Lyssavirus genus, two species were isolated in France. 47 positive bats were morphologically identified as *Eptesicus serotinus* and were shown to be infected by both the EBLV-1a and the EBLV-1b lineages. Isolation of BBLV in *Myotis nattereri* was reported once in the north-east of France in 2012. The phylogenetic characterisation of all 47 French EBLV-1 isolates sampled between 1989 and 2013 and the French BBLV sample against 21 referenced partial nucleoprotein sequences confirmed the low genetic diversity of EBLV-1 despite its extensive geographical range. Statistical analysis performed on the serotine bat data collected from 1989 to 2013 showed seasonal variation of rabies occurrence with a significantly higher proportion of positive samples detected during the autumn compared to the spring and the summer period (34% of positive bats detected in autumn, 15% in summer, 13% in spring and 12% in winter). In this study, we have provided the details of the geographical distribution of EBLV-1a in the south-west of France and the north-south division of EBLV-1b with its subdivisions into three phylogenetic groups: group B1 in the north-west, group B2 in the centre and group B3 in the north-east of France.

## Introduction

Bats represent about a fifth of the nearly 5000 known species of mammals with 1,232 bat species distributed worldwide and divided into two suborders: the Megachiroptera and the Microchiroptera versus Yinpterochiroptera and Yangochiroptera, respectively [1]. About 70% of bats are insectivores and most of the rest are frugivores. A few species feed on animals other than insects, one example being the hematophagous vampire bats. Bats are present throughout the world and play vital ecological roles in many ecosystems. Although much is known about the ecology and physiology of bats, they are also among the less studied mammals with regard to immunology [2]. Currently, more than 100 viruses have been isolated from bats [3]. Bats have been shown to be involved in several emergent viral diseases caused by coronaviruses, flaviviruses, astroviruses, adenoviruses, henipaviruses, filoviruses and lyssaviruses [4].

Rabies in bats is caused by negative-sense single–stranded RNA viruses of the genus lyssavirus, family *Rhabdoviridae*. Rabies virus is usually transmitted through the saliva of an infected animal during a bite. There are currently twelve recognised members of the genus lyssavirus [5]. These include classical *rabies virus (RABV), Lagos bat virus (LBV), Mokola virus (MOKV), Duvenhage virus (DUVV), European bat lyssavirus type 1 (EBLV-1), European bat lyssavirus type 2 (EBLV-2), Australian bat lyssavirus (ABLV), Irkut virus (IRKV), Aravan virus (ARAV), Khujand virus (KHUV), West Caucasian bat virus (WCBV)* and *Shimoni bat virus.* Three recent lyssaviruses have been identified but have not yet been taxonomically assessed: Ikoma bat lyssavirus (IKOV) discovered in 2009 in an African civet [6], Bokeloh bat lyssavirus (BBLV) identified in *Myotis nattereri* in Germany [7] and in France [8] and Lleida bat lyssavirus in *Miniopterus schreibersii* in Spain [9]. With the exception of IKOV and MOKV, bats are natural reservoirs for all lyssaviruses.

In Europe, bat rabies is principally caused by two rabies virus species, namely EBLV-1 and EBLV-2. EBLV-1 circulates widely throughout Europe with two variants, which are EBLV-1a and EBLV-1b. Variant a exhibits an east-west distribution from Russia to the centre of France with very little genetic variation while variant b exhibits a south-north distribution from Spain to Denmark and far more genetic diversity [10]. Most EBLV-2 cases were isolated in the United Kingdom (UK) and the Netherlands, and although EBLV-2 was detected in Finland, Germany and Switzerland, two countries neighbouring on France, up to now no cases of EBLV-2 have been reported in our country.

EBLV-1 is mainly associated with the serotine bat (*Eptesicus serotinus*) with 95% of the cases [11] and *E. isabellinus* in Spain [12] while EBLV-2 is reported mainly in Daubenton's bats (*Myotis daubentonii*) and pond bats (*Myotis dasycneme*). Single rabies cases have also been found in *Myotis myotis* and *Plecotus auritus* in Poland and occasionally in other bat species [13]. These include *P. nathusii* and *P. pipistrellus* in Germany, *R. ferrumequinum* in Turkey and *Nyctalus noctula in Yugoslavia* [*14*].

In France, bat rabies surveillance was initiated in 1989 after the discovery of the first positive case in a serotine bat in the north-eastern (NE) part of the country [15]. Since 2000, bat rabies surveillance has been improved with the consolidation of the network and involvement of local veterinary services in addition to the French national bat conservation network (SFEPM). As a result, the number of collected specimens has increased and data has enabled the estimation of rabies incidence in bat populations across the country.

In this study, we report the results of French passive surveillance of bat rabies from 1989 to 2013. We analysed the epidemiologic patterns of EBLV-1 in serotines from 1989-2012 using diagnostic laboratory data. Environmental (seasonal, annual, and geographical) and biological (sex) factors that could potentially affect the occurrence of rabies cases in serotines, were analysed in an attempt to improve knowledge of the dynamics of EBLV-1 infection. In addition, in the present study we undertook the phylogenetic analysis of the partial nucleoprotein gene from the collection of EBLV-1 samples with referenced sequences (GenBank database) isolated in Europe, to better understand the circulation of the two lineages of EBLV-1 in the country.

## Materials and Methods

### Data Source and laboratory diagnosis methods

The data analysed in the study included the passive bat rabies surveillance data collected from 1989 to 2013 by ANSES's Nancy Laboratory for Rabies and Wildlife, which is one of the two laboratories involved in rabies diagnosis in France. The national passive surveillance network is based on the testing of sick bats, bats suspected of having rabies (showing clinical signs or abnormal behaviour) or bats found dead (all species), for the detection of lyssavirus infections [16]. All dead bats found in human environments as well as sick and suspicious bats with no human contact were submitted for rabies testing at ANSES's Nancy laboratory. The data included the diagnostic results, information on the location where the bat was isolated, date of submission, morphological identification of the bat [17] and the name of the collector (local veterinary services or bat handler from the French national bat conservation network). Bat cases linked to human contamination, which are submitted to the Pasteur Institute in Paris, were excluded from the study.

The laboratory techniques used for routine diagnosis are the ones recommended by WHO and OIE, i.e. fluorescent antibody test (FAT), rabies tissue culture infection test (RTCIT) on murine neuroblastoma cells [18] and in cases of positive rabies diagnosis an additional RT-qPCR and typing of the isolated virus by Sanger sequencing [19].

The Fluorescent Antibody Test was performed on brain tissue specimens using polyclonal fluorescent isothiocyanate-labelled rabbit anti-rabies nucleocapsid immunoglobulin G (BioRad, France) [20]. The detection of infectious particles in brain homogenate was performed using RTCIT on neuroblastoma cells with an incubation period of 48 hours at 37°C as previously described [21]. The conventional hnRT-PCR was performed with universal rabies primers JW12-JW6 as previously described [19]. Evagreen RT-qPCR was performed using rabies primers [N165-N146 and JW12], 2 µl of cDNA generated from 5 µl of total RNA and the SsoFast Evagreen Supermix kit (BioRad, France) [22].

### Statistical analysis

The proportion of rabid bats was calculated according to different environmental and biological factors including the year the animal was submitted, season, region and sex. Prevalence comparisons were carried out using the Pearson's chi-squared test (χ2) or, as indicated by the Cochran rules, the Fisher exact test when a frequency was below 5 [23]. The 95% confidence intervals (95% CI) of percentages were calculated using free open-source R software, version 2.12.2 (R Development Core Team, 2011). Animals with undetermined rabies diagnosis status were excluded from the analysis.

### Phylogenetic analysis

A total of 49 rabies viruses (48 serotines and 1 Natterer's bat), obtained from the laboratory's virus collection were included in the phylogenetic study. Of the 49 tested samples, 48 bats (i.e. 47 serotines and 1 Natterer's bat), were isolated in France and 1 in Luxembourg (a serotine bat), a neighbouring country of France. All 49 of the samples studied were initially tested by the direct fluorescent antibody test prior to genetic characterisation. The analysed samples consisted of original frozen brain material (n = 42) and passaged brouse brain material (n = 7).

Viral RNA was extracted from 200 µl of a 10% brain tissue homogenate using an Iprep PureLink Virus kit (Invitrogen, France) according to the manufacturer's instructions.

The cDNA synthesis and PCR amplification of the partial nucleoprotein (N) gene were performed as previously described [19]. 5 µl of PCR products were separated on a 2% agarose gel then purified on Nucleospin Extract II columns (Macherey Nagel, France) and sequenced in both directions by Beckman Coulter Genomics (Takeley, Essex, United Kingdom). Consensus sequences were obtained by aligning the forward and reverse sequences with Vector NTI software (version 10.3.1). Duplicate sequences with a 100% match were removed from the dataset (n = 15). The N gene analysis was constituted of 52 sequences (390 nucleotides, positions 71 to 460; nucleotide numbering is based on the Pasteur virus genome NC_001542) including 32 serotine isolates from France, one serotine isolate from Luxembourg, the French BBLV isolate (KC169985), 13 representative EBLV-1 sequences (Spain, Germany, Poland, Netherlands, Russia, Ukraine, Denmark), 5 representative EBLV-2 strains (United Kingdom, Switzerland, Germany, Finland, Netherlands), the German BBLV (JF311903) and one referenced sequence (WCBV (EF614258)).

Phylogenetic trees were constructed using the Neighbour-Joining (NJ) method and the Maximum-likelihood (ML) method (both with the Kimura-2 parameter calculation) with MEGA software, version 5 [24]. The bootstrap probabilities of each node were calculated using 1,000 replicates for both methods. Bootstrap values over 70% were considered as statistically significant since the bootstrap method may be a conservative estimate for the reliability of a clade [25].

In addition, a Bayesian analysis of the nucleotide sequences including the 32 EBLV-1 isolates from France, the EBLV-1 isolate from Luxembourg, the only French BBLV isolate and 20 representative sequences was performed using the general-time reversible model (GTR, 10^7^ generations) of the molecular evolutionary software package Bayesian Evolutionary Analysis by Sampling trees (BEAST), updated to version 1.7. (http://beast.bio.ed.ac.uk) [26]. BEAST outputs (MCMC log files and tree files) were examined by using LogCombiner and TreeAnnotator (http://beast.bio.ed.ac.uk/Tracer), excluding the first 25% of trees from the analysis as burn-in. The phylogenetic tree was visualised by using FigTree (version 1.3.1; http://beast.bio.ed.ac.uk/FigTree). The scale bar indicates branch length, expressed as the expected number of substitutions per site.

Net p-distances between groups (N gene sequence, 390 nucleotides) were calculated by using MEGA 5 for the estimation of evolutionary divergence.

## Results

### Bat rabies surveillance

Between the inception of the bat rabies surveillance network in 1989 and 2013, 3176 bats were submitted for rabies diagnosis, 2447 of which were analysed. 1.96% (CI: 1.4–2.6) tested positive during this period. Of the 48 positive bats, 47 were morphologically identified as serotine bats (21 males, 16 females and 10 unsexed) and 1 as a Natterer's bat (female).

**Figure 1:**
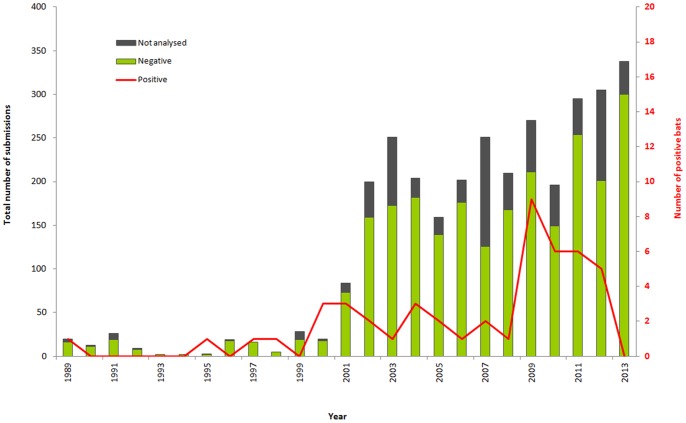
Changes in the number of dead autochthonous bats subjected to rabies diagnosis from 1989 to 2013.

Figure 1 represents the total number of autochthonous dead bats submitted for rabies diagnosis and the number of positive cases per year. From 2001 to 2013, a total of 3006 autochthonous dead bats were submitted for rabies diagnosis. Of the 3006 bats, classical rabies methods were not performed on 654 bats (654/3006 = 21.7%), due mainly to the high level of decomposition of the carcasses or to mummification. 41 bats were shown to be infected with EBLV-1 from 2001 to 2013 (Table 1), whereas only 7 positive cases were recorded from 1989-2000 (Figure 1, Table 2). The number of submissions increased 15 fold ( = 3006 versus 170) following the bat rabies surveillance reinforcement (Figure 1). This increase was accompanied by an increase in mummified bats (≈20% per year).

**Table 1:** Passive surveillance of bat rabies, 2001–2013.

Bats subjected to rabies diagnosis 2001–2013					
Family	Species	No. of mummified samples	No. of negative samples	No. of positive samples	Total no. of samples collected
Molossidae	*Tadarida teniotis*	16	6		22
Rhinolophidae	*Rhinolophus hipposideros*	7	41		48
	*Rhinolophus ferrumequinum*	4	30		34
	*Rhinolophus euryale*	2	7		9
Vespertilionidae	*Pipistrellus pipistrellus*	217	959		1176
	*Eptesicus serotinus*	60	217	40	317
	*Pipistrellus kuhlii*	8	203		211
	*Myotis myotis*	159	76		235
	*Pipistrellus nathusii*	4	115		119
	*Plecotus austriacus*	16	82		98
	*Miniopterus schreibersii*	5	36		41
	*Nyctalus leisleri*	7	64		71
	*Myotis emarginatus*	8	32		40
	*Plecotus auritus*	6	50		56
	*Pipistrellus sp*	53	108		161
	*Myotis daubentonii*	23	23		46
	*Myotis mystacinus*	3	30		33
	*Pipistrellus pygmeus*	8	39		47
	*Myotis nattereri*	2	13	1	16
	*Myotis sp*	6	13		19
	*Pipistrellus savii*		10		10
	*Myotis bechsteinii*	3	12		15
	*Nyctalus noctula*	5	76		81
	*Barbastella barbastellus*	4	19		23
	*Eptesicus nilssonii*	1	3		4
	*Plecotus sp*		4		4
	*Myotis blythii*		5		5
	*Myotis brandtii*		2		2
	*Vespertilio murinus*		2		2
	Myotis alcatoe		1		1
	*Nyctalus sp*	1	3		4
	Not identified	26	30		56
					
		654	2311	41	3006

Details regarding the number of negative and positive cases are given by family and by bat species.

**Table 2:** Passive surveillance of bat rabies, 1989–2000.

Bats subjected to rabies diagnosis 1989–2000					
Family	Species	No. of mummified samples	No. of negative samples	No. of positive samples	Total no. of samples collected
Molossidae	*Tadarida teniotis*				0
Rhinolophidae	*Rhinolophus ferrumequinum*	7	6		13
Vespertilionidae	*Pipistrellus pipistrellus*	6	25		31
	*Eptesicus serotinus*	0	16	7	23
	*Pipistrellus kuhlii*		1		1
	*Plecotus austriacus*		1		1
	*Myotis nattereri*	0	1	0	1
	*Nyctalus noctula*		2		2
	*Not identified*	14	84		98
		27	136	7	170

Details regarding the number of negative and positive cases are given by family and by bat species.

Of the 3006 submitted bats, 98% (n = 2950/3006) were morphologically identified by the laboratory or directly by the bat worker. 27 bat species were submitted for rabies diagnosis out of the 34 bat species reported in France, the pipistrelles being the most highly represented with 1176 samples (Table 1). The 27 collected bat species represented the three families of the order Chiroptera: *Molossidae* (n = 22), *Rhinolophidae* (n = 91) and *Vespertilionidae* (n = 2893 collected with 317 common serotines).

Improvements in the bat surveillance network were accompanied by a strong increase in pipistrelle bat submissions (56%), while the proportion of *E. serotinus* (10.5%) and *M. daubentonii* (1.5%) bats submitted did not vary.

**Figure 2:**
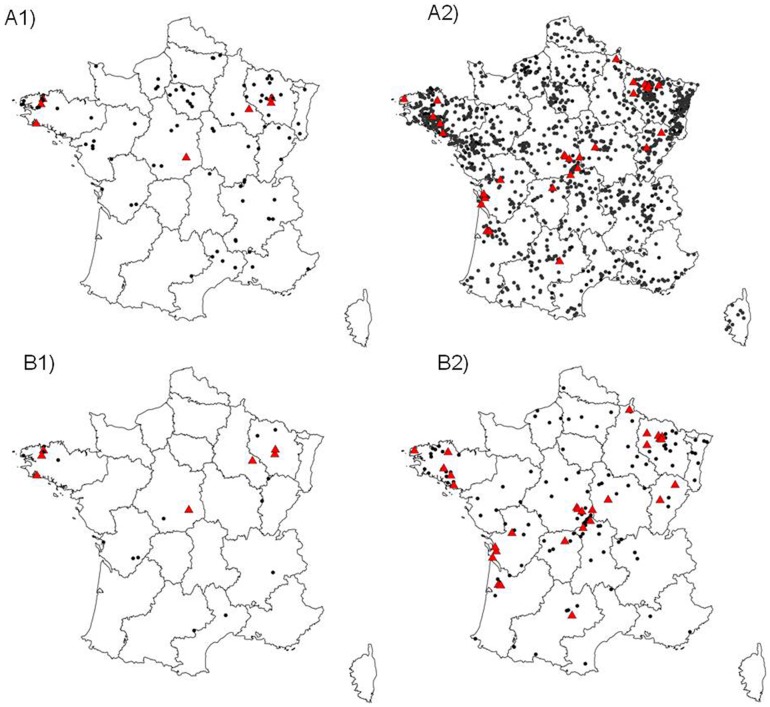
Bat rabies in France. Negative case: black dot, positive case: red triangle (A). Distribution of all the bat samples analysed for rabies diagnosis; A1)1989–2000; A2) 2001–2013 (B). Location of serotine (*Eptesicus serotinus*) samples analysed for rabies diagnosis; B1)1989–2000; B2)2001–2013.

Figure 2 illustrates the geographical distribution of all bats submitted for rabies diagnosis since 1989. While the samples submitted were from throughout the country, the number of bats sampled for rabies diagnosis varied by region (Figure 2A). In this study, the north-west (NW) regions were defined as Bretagne, Normandie and Pays-de-la-Loire, the north-east (NE) regions included Alsace, Champagne-Ardenne, Ile-de-France, Franche-Comté, Lorraine, Nord-Pas-de-Calais and Picardie, The centre regions (C) were defined as Auvergne, Bourgogne and Centre, the south-east (SE) regions included Languedoc-Roussillon, Provence-Alpes-Côte d'Azur, Rhône-Alpes and Corse, and the south-west (SW) regions included Aquitaine, Midi-Pyrenées, Limousin, Pays de la Loire and Poitou-Charente. Nearly half (44.7%) of the submitted bats came from the north-east of France, followed by the north-west (NW) (17.7%), the centre (C)(14.7%), the south-east (SE)(12.5%) and the south-west (SW) (10.5%).

**Figure 3:**
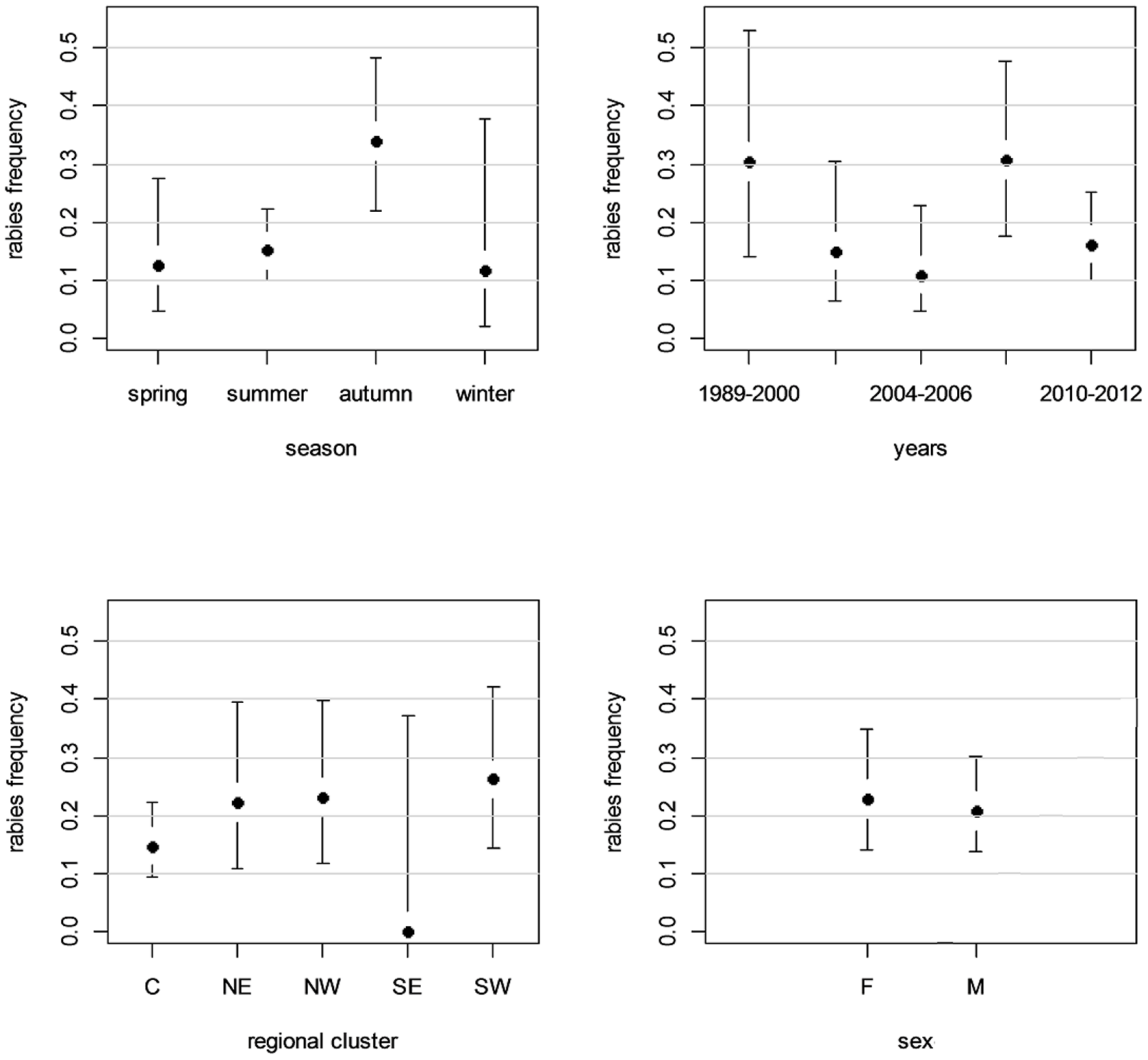
Rabies occurrence rate detected in serotine bats in France from 1989–2013 in the frame of the bat rabies surveillance network and according different environmental factors: season (a), year (b), regional cluster (c) and sex (d).

The analysis of the environmental (spatio-temporal) and biological (sex) factors that could potentially explain the occurrence of rabies in serotine bats was carried out. Temporally, the proportion of positive samples detected over 1989–2000 (30.4%; CI: 14.1–53.0) (i.e. before network reinforcement), 2001–2003 (15.0%; CI: 6.2–30.5), 2004–2006 (10.9%; CI: 4.5–22.9), 2007–2009 (30.8%; CI: 17.5–47.7) and 2010–2013 (16.2%; CI: 9.8–25.2) did not differ significantly (Figure 3a). When comparing rabies incidence during the different seasons (seasons defined as follows: Winter: December-February; Spring: March-May, Summer: June-August, Autumn: September-November), significant variations were observed (Fisher exact test, p = 0.005). Rabies cases were more frequent in autumn (34.0%; CI: 21.9–48.4) than in summer (15.1%; CI: 9.9–22.2) and in spring (12.5%; CI: 4.7–27.6) (Figure 3b). The test failed to detect any difference in rabies incidence between the winter (11.8%; CI: 2.1–37.8) and other seasons because of the low statistical power due to the low number of samples submitted between December and February.

The epidemiological data regions for the serotine were merged into NE, SE, C, SW and SE clustering regions to enable statistical spatial analysis. The probability of detecting rabies did not differ significantly by the geographical origin of bat sampling. Rabies prevalence was 26.2% (CI: 14.4–42.3) in C, 23.1% (CI: 11.7–39.7) in SW, 22% (CI: 10.7–39.6) in NW, 14.7% (CI: 9.3–22.3) in NE and 0% (CI: 0–37.1) in SE (Figure 3c).

When considering the sex of the animals, the probability of reporting a positive case in serotines did not differ between males and females, with respectively 22.9% (CI: 14.0–34.7) and 20.8% (CI: 13.6–30.4) of positive cases (Figure 3d).

### Phylogenetic analysis of the French EBLV-1 isolates

**Figure 4:**
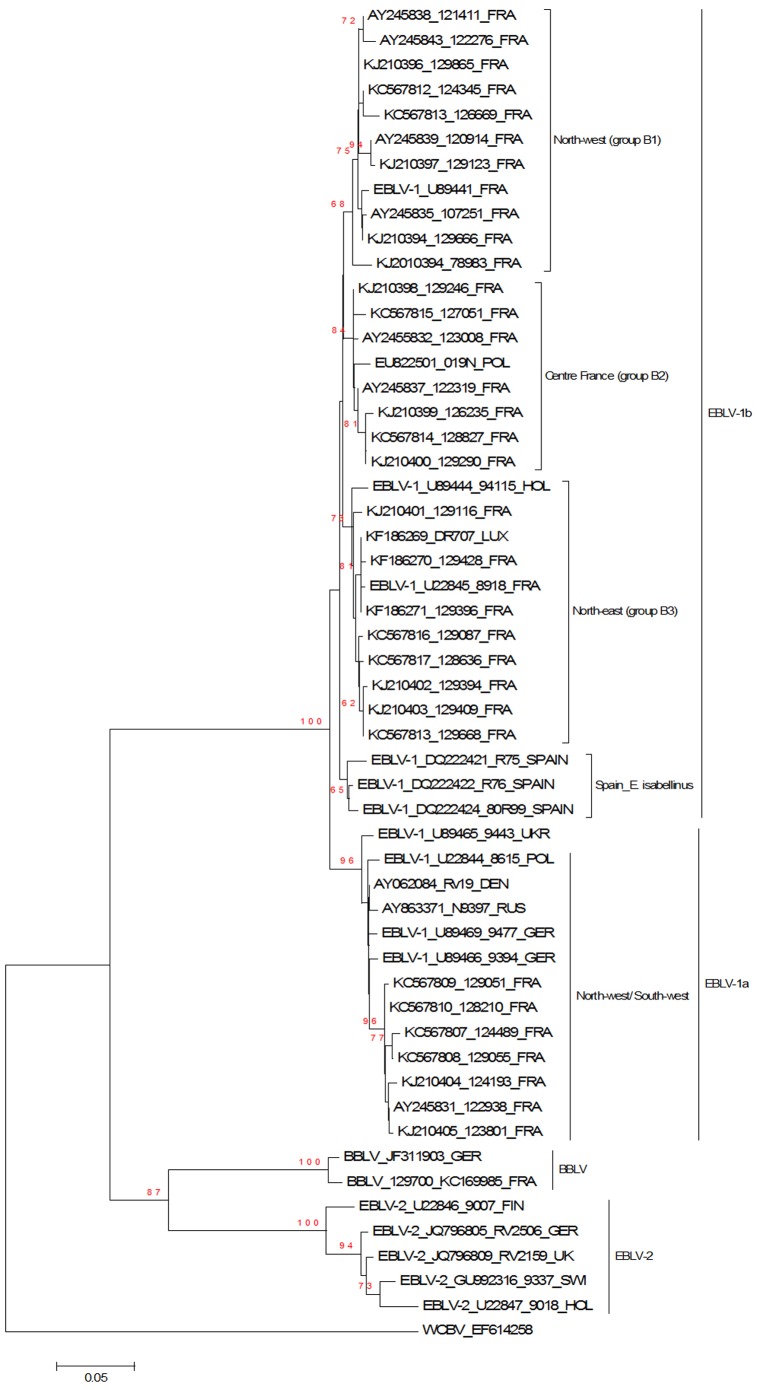
Neigbour-Joining and Bayesian Phylogenetics. A. NJ phylogenetic tree comparing partial N gene sequence from 32 French EBLV-1 isolates from 1989–2012, 11 referenced European EBLV-1 strains (Poland, Holland, Luxembourg, Spain, Denmark, Germany, Russia), 1 EBLV-1 isolate from Luxembourg, 5 EBLV-2 strains (Finland, Germany, United Kingdom, Switzerland, Holland) and two BBLV isolates (France, Germany). Relationships are presented as a rooted phylogram with WCBV (EF614258). Bootstraps above 70% are significant.

The phylogenetic study using the NJ method showed that all 32 infected serotines clustered with EBLV-1 viruses (bootstrap of 100). Figure 4 shows the results of phylogenetic relationships between the partial N gene sequence of the 32 French EBLV-1 sequences and 22 representative sequences. The Neighbour-Joining tree showed that the 32 French EBLV-1 strains belonged to the two lineages a and b of EBLV-1 (bootstrap of 100).

Cluster A forming the EBLV-1a lineage included 7 sequences from France and 6 sequences from Poland, Ukraine, Russia, Denmark and Germany while the EBLV-1b lineage (cluster B) was made up of 25 French samples and isolates from Luxembourg (n = 1), Poland (n = 1) and Holland (n = 1) (Figure 4). The 3 isolates from Spain belong to a distinct subgroup with a low bootstrap of 57%. The comparison between these 3 strains and the EBLV-1b cluster showed 97.1% nucleotide similarity. The nucleotide similarity was 98.6% among the three Spanish samples. The maximum likelihood method showed the same arrangements of clusters in the tree (data not shown), the EBLV-1b sequences from France being subdivided into three groups (with the bootstrap support above 70 for the three groups).

The first subgroup, B1 (bootstrap of 75), contained 7 strains isolated in the north-west of France and two strains from the centre of France (Isolates 107251 [AY245835] and 129666 [KJ210394]), respectively isolated in 1995 and in 2012 in the same city (Bourges, 47° 05′ 04″ north 2° 23′ 47″ east). One sample (78983 [KJ210395]), isolated in 1989 in the north-east of France, linked the subgroup B1 (limited bootstrap of 68). B2 (bootstrap of 84) was made up of 7 sequences from the centre of France and one referenced strain from Poland (EU822501). The last subgroup, B3 (bootstrap of 73), contained 8 strains from the north-east of France isolated between 1989 and 2012, one strain from Luxembourg isolated in 2013 (*E. serotinus*, KF186269) and one isolate from Holland (U89444, *E. serotinus* from 1992).

Cluster A (EBLV-1a) had less than 1.6% nucleotide divergence and 1% amino-acid divergence among all samples. In cluster B, 97.6% nucleotide similarity and 99.1% amino-acid similarity was shown among all sequences.

Bayesian phylogenetics with the BEAUti and the BEAST 1.7 software package showed the same division of the EBLV-1b lineage with the same geographical clusters throughout France: the centre, the north-west and the north-east (data not shown).

**Figure 5:**
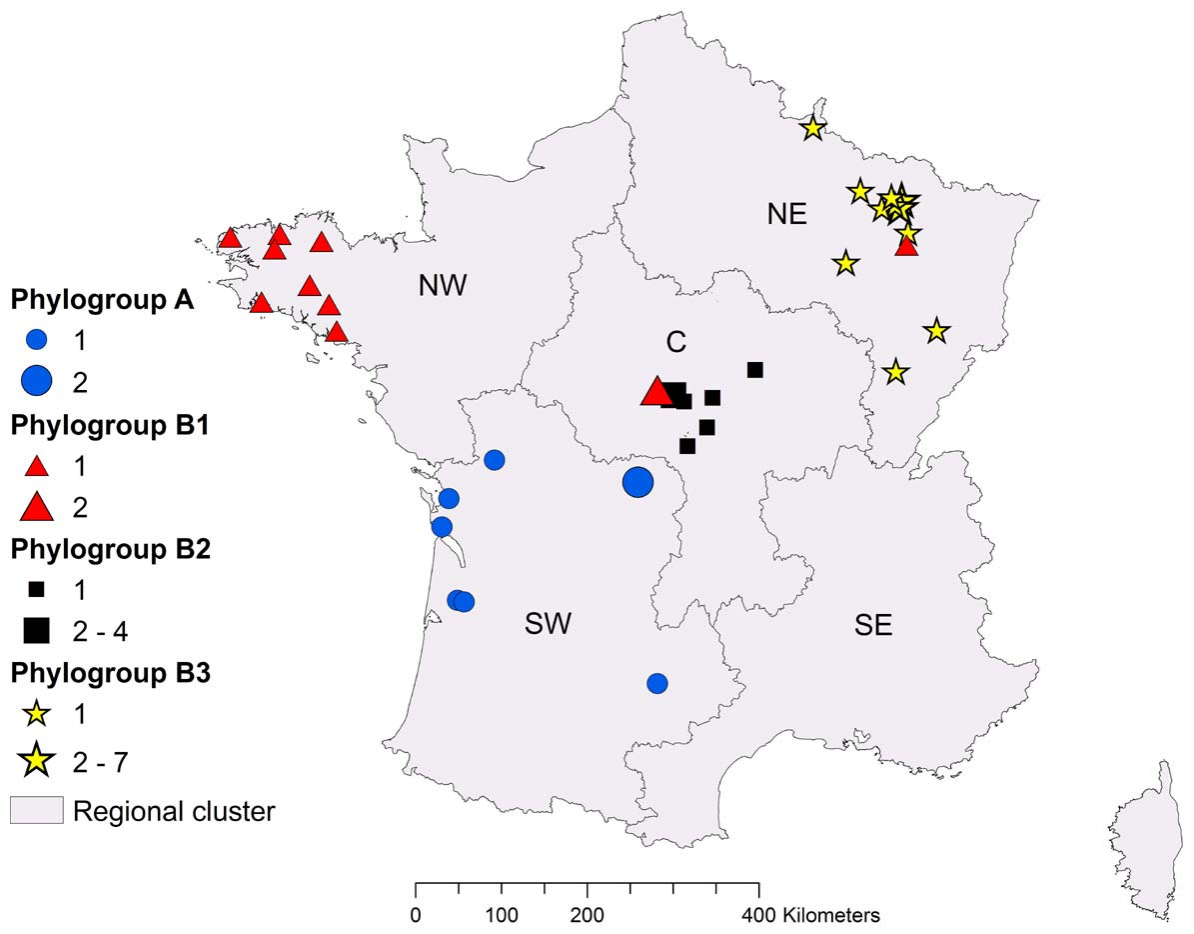
Regional clusters with geographical location of the 47 EBLV-1 a and b samples according to the results of the phylogenetic N gene analysis. Samples were located geographically according to the defined lineage: [B1 (north-west), B2 (centre), B3 (north -east)] and A (south-west)]. The numbers correspond to the identification numbers of isolates

Figure 5 shows a map of France illustrating the location of the 47 samples according to the results of phylogenetic analysis of the N gene showing the two lineages EBLV-1a (cluster A) and EBLV-1b (cluster B), as well as the three identified sub-groups, B1, B2 and B3. The subdivision of the French sequences is geographically clustered. Taking into account the 32 unique sequences used in the phylogenetic analysis and the 15 identical sequences (with respectively 13 and 2 isolates belonging to groups B and A), the subgroups B1 and B3, composed of 11 and 18 samples respectively appeared to be restricted to the NW (n = 8) and the NE of France (n = 18), respectively (Figure 5). 3 samples belonging to subgroup B1 came respectively from the centre (n = 2) and from the north-east (n = 1). The first positive case reported in the north-east of France (Bainville, KJ210395) in 1989 belongs to subgroup B1. Identically, the second case [AY245835] reported on 1995 in Bourges (centre of France) was also grouped in B1 with an additional case occurring in the same city (Bourges, KJ210394) on 2012. The 9 remaining samples belonging to subgroup B2 were located in the centre of France, with 6 samples in the Centre region, 2 in Auvergne and 1 in Bourgogne, while all EBLV-1a samples (group A, n = 9 samples) were located in the SW of France (Figure 5).

The estimation of evolutionary divergences using MEGA5 (p-distance calculation) showed an evolutionary distance of respectively 0.024, 0.014 and 0.005 for all French EBLV-1 a and b isolates and Spanish EBLV-1b.

## Discussion

France has been officially free of rabies in non-flying mammals since 2001 [27]. However, rabies remains a public health concern because of the risk of importation of infected animals [27] and the natural circulation of rabies in bats. Each year, around 4000 patients receive a post-exposure treatment. In 2011, of the 4150 patients receiving post-exposure treatment, 4.8% were due to bat exposure while over 79% were due to exposure to dogs and cats [28].

Since 1954, more than 1,000 bat rabies cases have been recorded in Europe (www.who-rabies-bulletin.org). Most cases were recorded in Western European countries with a well-established rabies network, particularly in the Netherlands, Denmark, Germany and Great Britain [13], where several cases are reported each year. The passive surveillance of rabies in bats is routinely based on the collection of dead bats for diagnostic testing.

Our study showed an increase of over 17 fold in the number of total bats submitted for rabies diagnosis in France between 2001 and 2013 (n = 3006 submissions) compared to the 1989–2000 period (n = 170 submissions). However the increase of the total number of samples is not accompanied by an increase in submissions of serotine bats and/or infected bats. In 2013, it is interesting to note an absence of reporting of positive case in the country despite the increase of total bats and despite the fact that all regions, particularly the Centre, Bretagne and Lorraine, where the majority of the cases were found, submitted serotine bats for rabies testing.

In Europe, *E. serotinus* is mainly found infected with EBLV-1 (more than 95% of cases [14]). Of the 43 bat species reported in Europe, a few other species were also shown to be infected with EBLV-1, including *Isabellinus serotinus* in southern Spain [29], and rarely, *Pipistrellus pipistrellus* (2 cases in Germany in 1987), *Plecotus auretus* (1 case in Poland), *Pipistrellus nathusii* (1 case in Germany in 1986), *Nyctalus noctula* (3 cases in Yugoslavia in 1954), *Myotis myotis* (1 case in Germany in 1973) and *Vespertilio murinus* (1 case in Russia in 1985) [13], [30].

Serotine bats are the only bat species shown to be infected with EBLV-1 in France. Up to 2013, EBLV-1 was the only virus species that had been detected in France, with the first case having occurred in 1989 in the north-east [30]. In 2012, BBLV was reported for the first time in *Myotis nattereri* in France thanks to effective collaboration with bat workers [8]. Our study showed, in conjunction with the increase of bat submissions, a high proportion of pipistrelles (≈40%), one of the most abundant bat species in France and also in southern Europe, over the period 2001–2013. All 1176 submitted pipistrelles were diagnosed negative for the presence of rabies, as previously reported in the Netherlands [31]. *P. pipistrellus*, which shows strong synanthropic behaviour, might be less exposed to lyssavirus [32]. Our results, in association with the philopatric behavior of this bat species could indicate, as previously suggested, that the risk of *P. pistrellus* infection with a Lyssavirus is almost non-existent [32] although its cohabitation with serotines is well known (Christine Harbusch, personal communication). The other bat species (*Plecotus auritus*, *Nyctalus noctula)* occasionally found to be infected with EBLV-1 were also found negative in our study, demonstrating that these tree-dwelling bats are also rarely infected with EBLV-1.

As previously reported in European countries having a well-established bat rabies surveillance network [31], [33], the collection of bats may be potentially biased due to the sampling scheme. The studied samples are not representative of the natural population of bats due to the small numbers of bat submissions. Compared to the total bat population, which includes millions of individuals, the submissions in the passive surveillance do not reflect the true natural proportion in the field. Moreover, certain species are underrepresented: of the 34 bat species reported up to now in France, 27 species were collected from 2001 to 2013 and only 7 from 1993–2000.

The apparent proportion of 1.96% (48/2447 analysed *100) infected serotine bats is certainly biased and does not reflect the true prevalence and the correct epidemiological bat rabies situation in France. In addition, passive surveillance could lack sensitivity due to the small numbers of serotine bats submitted for rabies diagnosis compared to the estimated numbers for this species in France (400,000–600,000) (Laurent Arthur, personal communication). Furthermore, high fluctuations in the numbers of bat submissions were observed between geographical areas. For example, in the centre of France, in an area of approximately 39,151 km^2^ [the total area of France is 552,000 km^2^], 7 serotine bats were found to be infected out of a total of 21 tested serotines and out of 48 positive cases. Up to now, 140 colonies of serotine bats have been reported in this region out of a total of 987 colonies housing other species [34]. While serotines are shown to be distributed throughout the country [35] and while some aspects of serotine bat ecology have been studied, including diet, habitat use, foraging and roosting behaviour, others are mostly unknown, especially its hibernation ecology, mating system, dispersal and population structure. An understanding of the spatial ecology, behaviour and social dynamics of serotine bat populations is essential for a better understanding of EBLV-1 prevalence patterns in bat colonies.

Previous studies showed that the incidence of rabies in bats submitted for rabies diagnosis exhibited a strong seasonal pattern with two peaks, in the spring and autumn [36], [37]. This seasonality has recognized importance for epizootiological processes [38]. The infection status of bats has already been shown to be affected by season for some viruses, including lyssaviruses, henipaviruses, coronoviruses and filoviruses [2]. Using passive surveillance data, we report for the first time in Europe the seasonality of the rabies incidence rate in bats, with a higher rabies incidence detected in autumn (September to November) reaching 34%. In our study, 81% ( = 38/47) of positive cases were detected between June and October. The highest probability of detecting a rabies case among submitted bats in autumn rather in summer could be explained by the natural dispersal of serotine bats which promotes the contact rates between males/females, adults/juveniles and between infected and susceptible animals. Dispersal is a poorly understood event in the serotine bat species, as is the mating period and the ecology of males. No adult males are found within maternity roosts, indicating exclusion once they reach sexual maturity [39]. The mating period, taking place in late summer/early autumn (August-October), could also promote the diffusion of EBLV-1 in bats.

As previously suggested [37], the interaction of the incubation period with hibernation and the annual birth pulse, which starts for serotines in early June but which depends on the climate, can generate complex dynamics for the rabies virus. To date, current knowledge about rabies in serotines in Europe is limited. Little is known about the virulence, dissemination or transmission of the virus among free-living insectivorous bats, or abouts the incubation period of European bat lyssaviruses. Although the incubation period is not known for bats naturally infected with EBLV-1, experimental EBLV-1 studies of *E. serotinus* and *E. fuscus* with an inoculation dose of 10^3.2^ LD50/ml showed that the incubation periods varied depending on the route of inoculation, and were respectively between 7 to 26 days and 9 and 58 days [40], [41]. By comparison, the duration of rabies incubation is also highly variable in bats naturally infected with classical rabies virus, ranging from less than 14 days to over 290 days, and can rarely be greater than a year [42], [43]. These variable incubation periods could also explain the temporal dynamics of rabies incidence in our study. Indeed, several previous studies have shown a higher seroprevalence in summer. Seasonal variation in European bat infection was observed through the analysis of a large blood sample panel in Spanish bats showing a significantly higher seroprevalence of EBLV-1 in the summer, at which time maternity colonies are present in most of the bat colonies [32]. This seasonal variability was also shown in Brazilian free-tailed bats in natural cave roosts [44], indicating that roosting ecology and reproductive activity could favor bat infection. This higher seroprevalence in summer reflecting higher rabies exposure could, depending on the incubation period, lead to a higher rabies mortality rate following RV exposure in the subsequent months, and mainly in autumn. The higher seroprevalence reported in summer could also be the result of the higher metabolism of serotine bats in summer than in winter and spring.

In Europe, bat rabies is caused by EBLV-1 and EBLV-2. On a few occasions, WCBV and BBLV have been reported [11]. To date, despite the strengthening of passive surveillance, neither EBLV-2 nor WCBV have been isolated in France although EBLV-2 was isolated in three neighbouring countries, i.e. Germany, the United Kingdom and Switzerland. (Reviewed by [45]). Two bat species were shown to be infected by EBLV-2, Daubenton's bat (*M. daubentonii*) and the pond bat (*M. dasycneme*). Of the two species, *M. dasycneme* is reported in the north-west of France, while Daubenton's bat is present throughout France. The absence of EBLV-2 reporting in France has yet to be explained. The number of submissions of *M. daubentonii* should be increased to provide more elements on the true epidemiological situation of EBLV-2 in France. The real absence of this virus species needs to be proven in the country.

In almost all animals, rabies is a fatal disease once clinical signs manifest. However, most bats are able to coexist with viruses and may have evolved mechanisms to control viral replication [2]. It is clear that bats can experience either a lethal or non-lethal rabies virus infection, with in the latter case the development of specific neutralising antibodies [46]. We previously reported the presence of EBLV-1 neutralising antibodies in six indigenous bat species throughout France, mainly in *E. serotinus* and *M. myotis*, suggesting previous infection with the EBLV-1 rabies virus [47]. However, no EBLV-1 RNA was detected in the RT-PCRs performed on oropharyngeal swabs collected from these six apparently healthy bats [47]. Similarly, a high percentage of seropositive bat species (65%) was recently found in Spain, suggesting that most species of bats are exposed to EBLV-1 [32].

EBLV-1 is widely distributed throughout Europe. Serotines infected with the EBLV-1a variant exhibited an east-west distribution from Russia to France with very little genetic variation [10]. Most cases of EBLV-1a were reported in France, the Netherlands, northern Germany, Denmark and Poland. EBLV-1b, which presents far more genetic diversity and a south-north distribution, was mainly reported in Spain, France, Germany, Poland and in the Netherlands [13]. Our study demonstrated that all French EBLV-1 strains belong to the two lineages, a and b, of EBLV-1. Serotines infected with EBLV-1a clustered geographically in the south-west of France while serotines infected by EBLV-1b appeared to be grouped in the centre of France, the north-west and the north-east. Surprisingly, serotines infected by EBLV-1a were geographically separated from the regions displaying EBLV-1b cases. This question of geographic separation has not yet been explained. One hypothesis is that serotines might be a paraphyletic group composed of differentiated lineages at species level, like the Natterer's bat [48], one species being specific to lineage a and the other species specific to lineage b.

Phylogenetic analysis confirmed the high variability of EBLV-1 with a complex phylogenetic structure for EBLV-1b separated into at least 4 lineages, in contrast to EBLV-1a which has low genetic diversity and only 1 lineage despite its extensive geographic distribution from France to the Netherlands and Russia.

It was successively recommended by the French expert working group on rabies in bats (AFSSA, 2003), the French Veterinary Academy (2005) and more recently by a group of European rabies experts [16], that research work and epidemiological surveillance of EBLV-1 infection be pursued and intensified. French passive bat rabies surveillance in particular should be improved with regard to targeted bat species such as serotines, Natterer's bats and Daubenton's bats. These results, complemented by active surveillance data [47], will help clarify the dynamics of EBLV-1 transmission and improve understanding of the cross-species infection process. To date, two cases of natural transmission of EBLV-1 to domestic cats have been reported in France [49], demonstrating that the two subtypes, a and b, of EBLV-1 are able to cross the species barrier. This clearly implies that bat biologists, virologists, and public health and agricultural service officials must work together closely in order to protect the public and promote bat conservation.
